# Optical remote sensing for monitoring soil erosion in sub-Saharan grassland biomes: a systematic review

**DOI:** 10.1007/s10661-025-14426-3

**Published:** 2025-08-01

**Authors:** Nyasha Mubonderi, Alen Manyevere, Chuene Victor Mashamaite, Mohamed A. M. Abd Elbasit

**Affiliations:** 1 Department of Agronomy, University of Fort Hare, Private Bag X1314, Alice, 5700 South Africa; 2 Arid Region Water Research Centre, Sol Plaatje University, Kimberley, 8300 South Africa

**Keywords:** Grassland monitoring, Gully expansion, Remote sensing, Land degradation, Topological factors, Vegetation cover

## Abstract

The Sub-Saharan African region is experiencing the effects of climate change and rapid population growth. The current population, together with the impacts of climate change, has a negative effect on soil resources; hence, implementing land conservation and sustainable land management methods is essential throughout the region. Policymakers require spatial information on soil erosion hotspots to make decisions because soil erosion has high negative impacts on agricultural lands. The study aimed to systematically review the literature on integrating optical and Synthetic Aperture Radar (SAR) systems and multiplatform satellite-based systems to monitor soil erosion in sub-Saharan grassland biomes. The review followed the PRISMA guidelines, and a bibliometric analysis was conducted to identify and evaluate relevant studies. In this study we include thirty-four articles for data extraction. Data was extracted and evaluated based on the techniques used to monitor soil erosion. The study reveals that the development of gullies varies and is closely linked to topography, specifically river flow networks and slope gradients. The findings highlighted the applications of different remote sensing techniques for monitoring soil erosion in the grasslands of sub-Saharan Africa. There is limited research on the effectiveness of SAR sensors for detecting the progression of soil erosion in grasslands, and there is insufficient validation of SAR-derived erosion models with ground-truth data in sub-Saharan Africa.

## Introduction

Land degradation significantly affects grassland biomes and is recognized as a major global challenge in the twenty-first century, necessitating urgent mitigation efforts (Eswaran et al., 2019). It involves nutrient depletion, compaction, acidity, biodiversity loss, and soil erosion (Gomiero, 2016). The rapid changes in land use and the shift towards more intensive cropland management practices are primary causes of land degradation (Corbeels et al., 2020). Overgrazing, non-adoption of adequate conservation measures of soil, waterlogging, seasonal rainfall, floods, and drought are some accelerating factors contributing to land degradation. Land degradation also threatens global food security, and as such, sustainable ways to reduce it have been implemented (Gomiero, 2016). To reverse land degradation, the United Nations declared urgent action in 2012 (Kust et al., 2017). Subsequently, the United Nations introduced Sustainable Development Goal 15 of Life on Land, which focuses on “protecting, restoring, and promoting the sustainable use of terrestrial ecosystems; sustainably managing forests; combating desertification; and halting and reversing land degradation and biodiversity loss” (United Nations, 2020).

Soil erosion is a huge danger to world food supply. It affects water quality, crop yields, atmospheric carbon dioxide concentration, reservoir sedimentation, and hydrological regimes, including increased flood risk (Locatelli et al., 2022). Globally, Asia ranks first, followed by Africa, in the severity of soil erosion (Karamage et al., 2016). The main contributors to soil loss are strong winds, heavy rain, and surface runoff. Soil erosion due to water accounts for much soil loss, amongst other factors. Wang et al. (2021), found out that soil loss due to water was estimated to be 1150 t/km^2^ in 2003. Due to financial constraints, lack of awareness, and climate challenges, farmers in many developing nations fail to replenish appropriately lost soil nutrients, and this makes their land more susceptible to erosion (Benavidez et al., 2018).

Many factors influence soil erosion, including vegetation conditions, climate, hydrology, topography, and human influence (Corbeels, et al., 2020). Soil erosional rates in sub-Saharan Africa vary. In East African countries like Tanzania, drought is a commonly encountered phenomenon, and there is high-intensity livestock production, which lowers soil cover, reducing the stability of soil aggregate, and reduced infiltration rates, resulting in more soil loss (Blake et al., 2021). However, the south-eastern coast of Southern Africa receives high rainfall with high intensity, which results in high soil losses (Molekwa, 2013). Tully et al. (2015), reported an estimate of 280 million metric tons of soil loss per year for sub-Saharan Africa.

Grasslands are vital ecosystems that play crucial roles in biodiversity conservation, carbon sequestration, and supporting livelihoods across the sub-Saharan Africa region. However, monitoring and understanding the functions of these biomes, specifically in the wider perspective of environmental crisis and land use change, remains challenging. Grasslands are among the most prevalent ecosystem biomes, and they contribute approximately 40.7% of the world's terrestrial surface, along with forests (Soubry et al., 2021). Many ecosystem services are provided by grassland, which include the regulation of climate, cycling of nutrients, provision of forage material, and habitat (Bengtsson et al., 2019). According to Millennium Ecosystems Assessment, human activities also lower the capacity of the ecosystems, including grasslands, due to human-induced fires and population expansion (Liu et al., 2021; Millennium Ecosystem Assessment, 2005).

Given these impacts of soil erosion on the environment and agricultural lands, policymakers require spatial information on soil erosion for decision-making. Monitoring erosion by its spatial extent is significantly important for effective preventing and addressing erosion effectively. Precise soil maps that offer detailed information about soil properties and their geographic distribution are essential for good land management of resources, land suitability assessment, and land evaluation (Minasny & McBratney, 2008). Monitoring soil erosion also helps determine the amount of soil lost in each area.

Monitoring of soil erosion in ecosystems has been done using remote sensing and Geographical Information System (GIS) (Soubry et al., 2021). Remote sensing devices play an important role in collecting data on the available moisture in the soil, topography, climate variables, temperature, crop growth, and vegetation indices. These inputs are essential for estimating soil erosion rates indirectly; they are used to get the inputs for soil erosion models such as the Revised Universal Soil Loss Equation (RUSLE) and the Universal Soil Loss Equation (USLE) to estimate soil erosion. These sensors, which can be deployed on various platforms like drones and satellites, enable the precise measurement of key variables that are essential for monitoring agricultural and environmental conditions (Thorp & Tian, 2004). These tools can detect rangeland properties which include topography, surface roughness, landscape, bare soil coverage, and vegetation patterns. The GIS techniques are employed to analyze and manipulate geospatial data (Soubry et al., 2021). These GIS techniques are also useful in integrating data from different sources, such as satellite images, climate data, soil data, and socioeconomic data.

According to Garg et al. (2020), the global demand for a systematic global soil resource database is increasing due to the growing global challenges in land management. A systematic review on the integration of different remote sensing data, like fusing optical and synthetic aperture radar (SAR) data within a multiplatform satellite-based framework focusing on grassland biomes in Sub-Saharan Africa is crucial due to several significant gaps in current research. Due to the need for technical expertise and the lack of infrastructure for processing SAR data, most studies in sub-Saharan Africa have not integrated optical and SAR data, leaving a critical gap in achieving more accurate and timely assessments of soil erosion. By synthesising existing literature through a systematic review, this study aims to systematically review the literature on optical remote sensing for monitoring soil erosion in sub-Saharan grassland biomes. Moreover, the methodologies and techniques employed in the development of multiplatform satellite-based systems for monitoring soil erosion using remotely sensed data from diverse platforms in sub-Saharan African grasslands are assessed.

## Methodology

This review employed a systematic mapping approach to gather and analyse existing scientific literature on the use of remote sensing techniques for monitoring soil erosion progression in degraded grasslands across sub-Saharan Africa. The systematic mapping approach was chosen to provide a comprehensive overview of the methods, technologies, and geographic distribution of studies, as well as to identify key research gaps in the monitoring of soil erosion using satellite based remote sensing. The study included all types of degraded grasslands, such as pasturelands, savannas, and rangelands, and examined soil erosion progression across various temporal scales, including annual, decadal, and long-term trends. The Preferred Reporting Items Systematic reviews and Meta-analyses (PRISMA) were used to establish the eligibility and the exclusion criteria in this study and for data abstraction and analysis. The systematic review was guided by PRISMA because it helps in assessing as much relevant and available scientific literature as possible in a defined time frame (Mengist et al., 2020). PRISMA demonstrates the quality of the systematic review, and it allows readers to evaluate the weaknesses and strengths of the review (Moher et al., 2010).

The Web of Science, Scopus, IEEE Xplore, and Google Scholar databases were searched for peer-reviewed journal articles published from 1990 to February 2024. Web of Science and Scopus provide comprehensive coverage of high-quality journals, IEEE Xplore includes numerous journals on technical advancements in remote sensing, and Google Scholar offers broader literature coverage. Hence, these databases were selected and used in this study to ensure a balanced and robust representation of soil erosion studies using remote sensing techniques. The year 1990 was selected as the starting point because it was a turning point when satellite-based remote sensing like Landsat and SPOT became widely used for environmental monitoring. Also, in 1990 there was an increase in global attention on land degradation, especially in sub-Saharan Africa, thus allowing this study to thoroughly examine how methods for studying soil erosion have evolved over the past three decades. The keywords combinations used in the Web of Science and Scopus databases are as follows: Soil degradation” OR Land degradation” AND “Soil erosion” OR"Soil loss” AND “sub-Saharan Africa” AND “Sustainable Development Goal’’ AND “Remote sensing” AND “Geographic Information System” AND “Synthetic Aperture Radar Systems” AND “Grassland biomes” OR “Land use” OR “Land practice” AND “Abiotic Factors” AND “Climatic Factors” AND “Topological Factors” AND “Control measures” OR “Control strategies” AND “Vegetation density” OR “Vegetation cover” AND “Sheet erosion” AND"Gully erosion” AND"RUSLE” AND"USLE” AND"Catchment area” AND"Aster data” AND"Land cover changes”.

### Selection criteria

The systematic searching was done in stages. The initial stage was to identify key words that were obtained from previous studies on soil erosion monitoring. This was followed by a screening stage where duplicates were removed, and the eligibility and exclusion criteria were conducted. The articles included in the study concentrated on monitoring soil erosion using remote sensing methods. In grasslands and catchment regions, the application of soil erosion models for monitoring is linked to factors such as rainfall, wind, animal activities, and topography within the sub-Saharan African area. The studies used in this study were researched according to predefined selections summarized in Table 1 below.

**Table 1:** Overview of the selection criteria applied in the study

Criterion	Inclusion Criteria	Exclusion Criteria
Article Type and Language	All articles, notes, survey papers, and conference papers written in English	All articles, notes, survey papers, book chapters, and conference papers not written in English
Countries or Region	Studies conducted inside sub-Saharan Africa were included	Studies conducted outside sub-Saharan Africa
Timeline	Between 1990 and 2023	< 1990
Study Design	Studies with adequate systematic approaches were included	Studies with inadequate systematic approaches were excluded
Monitoring Technique	All studies include remote sensing techniques	All studies that excluded remote sensing techniques
Land Use	Studies conducted in grasslands and catchments	All studies that were not conducted in grasslands and catchments
Data Quality	Qualitative studies that are well reported	Studies with poor data quality were excluded
Peer Review	Peer reviewed articles	The current study excludes non-peer reviewed articles

### Data extraction

Articles were screened based on the time when the article was published, the study design used, the study region, peer review status and the quality of the data in the articles identified. The articles used in the review were all written in English, peer reviewed articles and these studies were conducted in grasslands and catchment areas within the sub-Saharan Africa region. After the screening process, the data was extracted from the included articles.

### Data analysis

The results were presented in tables, and descriptive statistics were illustrated using bar graphs. For data management, Excel and Mendeley software were employed to acquire and store data from the databases, and duplicates were eliminated. The Mendeley software was used because it can import and arrange references, create bibliographies, organize sources using groups, tags, and filters, and allows the author to collaborate with others (Banat, 2021). A bibliometric analysis was conducted to identify emerging trends and key research themes, focusing on author keywords from documents that met the eligibility criteria. This approach provided insights into the most frequently studied topics, the evolution of research focus over time, and areas requiring further investigation in the field of soil erosion monitoring using remote sensing techniques. The VOSviewer version 1.6.16 was utilised to visualise the relationships and coherence among the authors'keywords.

### Risk of bias assessment

The Crowe Critical Appraisal Tool was utilised to evaluate the review methodology, focusing on key criteria that include preliminaries, introduction, design, sampling, data collection, ethical considerations, results, and discussion and conclusion (Kadir Shahar et al., 2020).

## Results and discussion

### Study selection

**Fig. 1:**
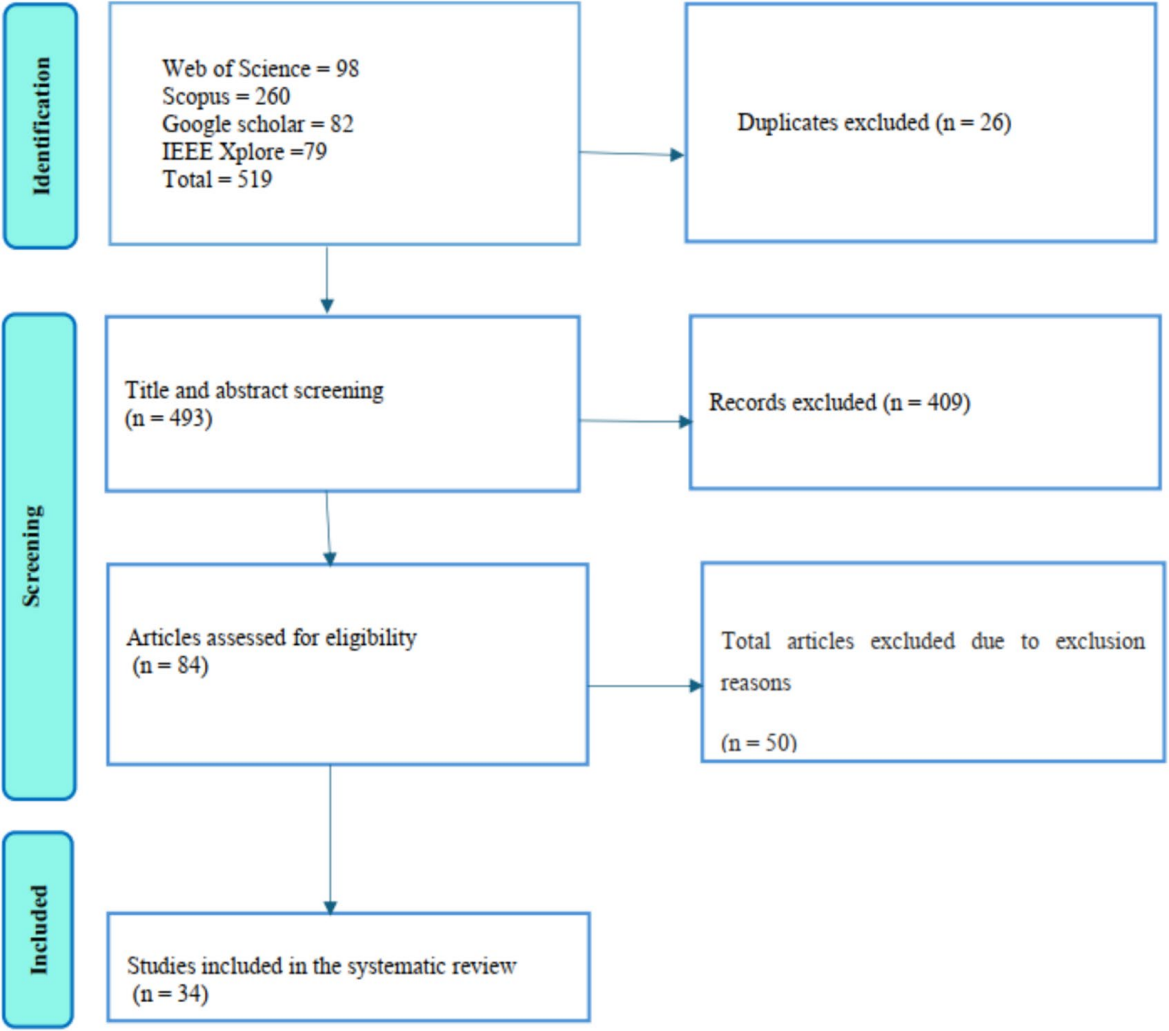
Flow diagram of the selection process used to identify studies for inclusion based on Preferred Reporting Items Systematic reviews and Meta-analyses guideline. The letter n = number of articles

The study identified 519 records from four databases, removed 26 duplicates, and reconstructed 403 records. The 84 articles qualified for full-text review, with 50 excluded due to exclusion reasons which include non-grasslands, pre-1990, or traditional methods. The 34 articles met the selection criteria. Figure 1 below illustrates the selection process used to identify studies for inclusion based on PRISMA guidelines.

### Geographic distribution and publication trends

**Fig. 2:**
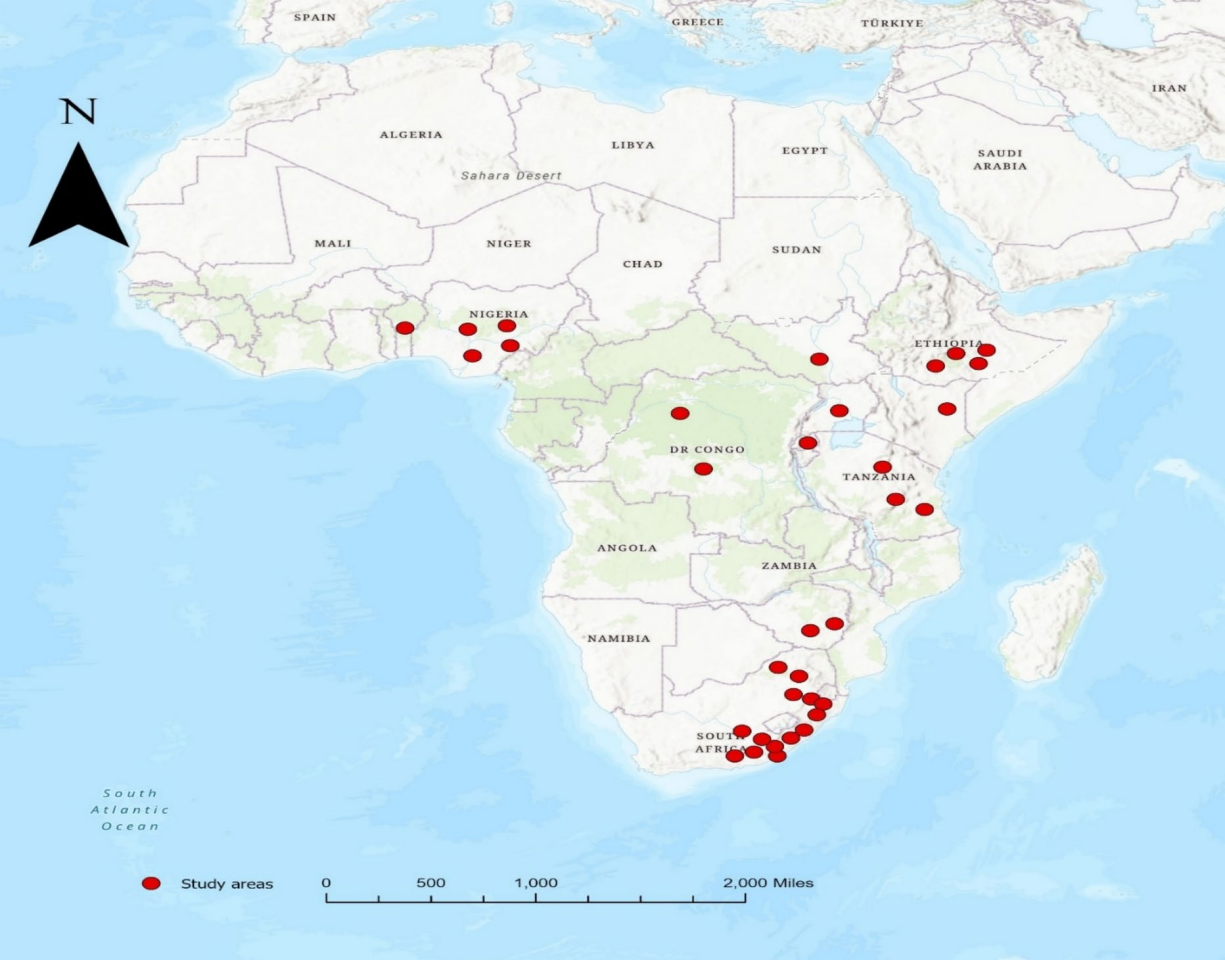
The geographical distribution of the studies has been included in the systematic review

This study included 14 studies conducted in South Africa, mainly in Eastern Cape, KwaZulu‒Natal and Limpopo, provinces. The other studies included were carried out in Zimbabwe (n = 2), Eastern Sudan (n = 1), Tanzania (n = 3), Kenya (n = 1) Rwanda (n = 1), Ethiopia (n = 4), Uganda (n = 1) Republic of Benin (n = 1), Democratic Republic of Congo (n = 2), and Nigeria (n = 4). Figure 2 shows how the studies included are frequently published. Figure 2 below shows the geographical distribution of the studies included in the systematic review.

**Fig. 3:**
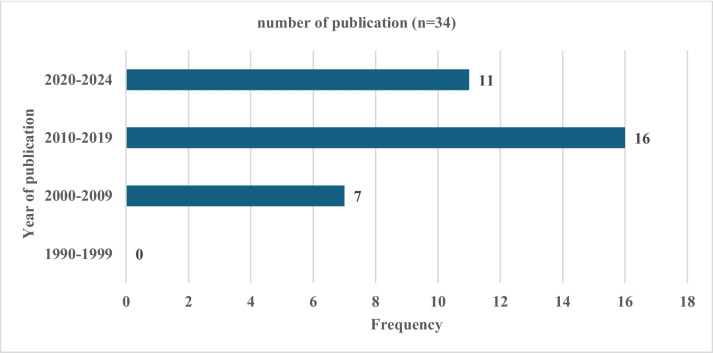
The frequency of included studies published from 1990 to 2024 conducted within the sub-Saharan African region

The study included no publications from 1990 to 1999 since none of the screened articles fit the inclusion criteria. As sub-Saharan African nations'technological capabilities improved between 2010 and 2019, more publications that employed the technology were published (Kulimushi et al., 2021a, 2021b). Compared with the articles from 2010 to 2019, there are fewer from 2020 to 2024 because the monitoring processes take time, methodological and technological adaptation delays, and COVID-19-related disruptions. Particularly in the present, there are noticeable gaps in the literature regarding remote sensing techniques, particularly multiplatform satellite-based systems for monitoring soil erosion in sub-Saharan African grassland biomes (Fig. 3).

### Bibliometric analysis

**Fig. 4:**
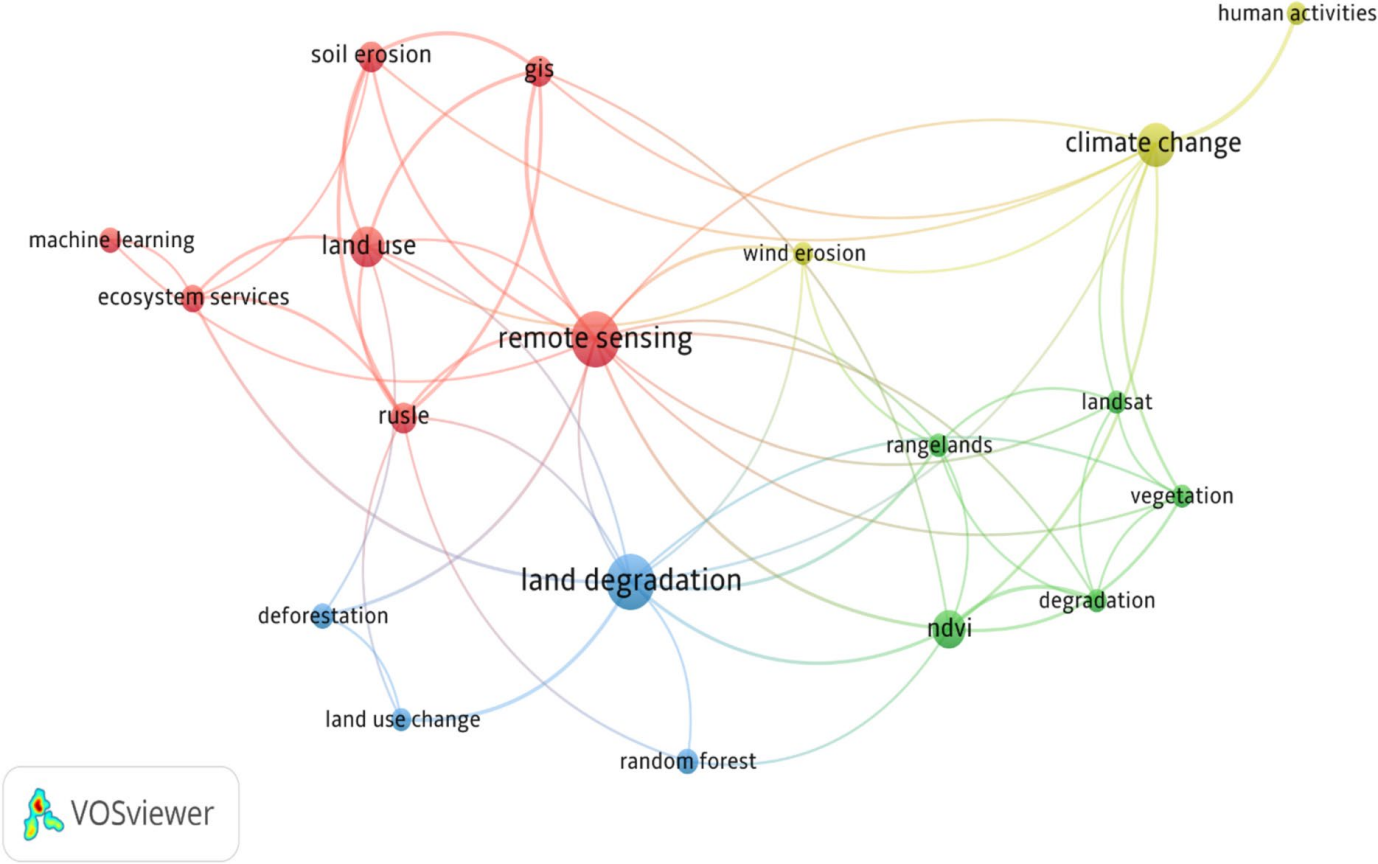
A bibliometric analysis of the author keywords

Figure 4 shows a bibliometric analysis of the author keywords from the titles and abstracts of the articles qualified and eligible for review. The coherence of keywords, and the node size indicates the frequency of occurrence, and their co-occurrence is represented by the curves between nodes. If there is a short distance between two nodes, it means there is high coherence between the two words. Soil erosion is represented by five clusters, with 161 links and a total link strength of 99. This shows that soil erosion was strongly connected to several key topics such as remote sensing, land use change, land degradation and ecosystem services. Remote sensing has one cluster, with 15 links and a total strength of 24 with an occurrence of 29. It was primarily linked to topics like land cover, GIS, land degradation, ecosystem services, and soil erosion. The results indicate that remote sensing was commonly associated with technologies and methods to monitor and examine land degradation, grassland ecosystems, and the changes in land cover, and soil erosion studies. Remote sensing provides important spatial data on land cover changes, ecosystem health, and soil erosion patterns. Land use changes, such as agricultural expansion and deforestation, are significant drivers of soil erosion (Corbeels et al., 2020). These activities remove the protective vegetation cover that stabilises the soil, leading to reduced soil stability and increased susceptibility to erosion.

### General characteristics of the included studies

Multiplatform satellite-based methods were employed in the selected studies to monitor soil erosion progression in sub-Saharan grassland biomes. The GIS and soil loss models were also applied together to monitor the progression of soil erosion. All 14 included studies were conducted on a large scale within the sub-Saharan grassland biomes. Gullies are mainly dominant in the grassland biomes, and multispectral satellite-based images are highly utilised for monitoring gullies because they are more accurate and efficient than traditional methods (Elachi & Van Zyl, 2021). Multispectral satellite-based images have higher resolution, wider coverage, temporal monitoring capabilities, cost and time efficiency, and they have ability to identify non-visible features as compared to traditional. Only one study focuses much on landslide mapping (Odhiambo et al., 2019). Three selected studies focus on grasslands within the catchment areas (Mhangara et al., 2012; Muhoyi & Muhoyi, 2023; Nzuza et al., 2021).

### Main findings

#### Use of multiplatform satellite-based in monitoring soil erosion

Table 2 illustrates the main findings for each of the articles included. The selected articles reported that the use of multiplatform satellite-based systems in monitoring soil erosion in sub-Saharan grassland biomes has increased from the early 2000 s to the present. However, they have some limitations which include cloud cover mainly in tropical regions, and in some areas, they are restricted to access higher resolution, and this hinders their effectiveness (Dzurume et al., 2022). The results from the selected articles showed that in monitoring soil erosion progression, multispectral Landsat series, Sentinel 2 satellite, Landsat 8, ASTER, SRTM, GIS, soil loss models, and field validation through field surveys are employed. The selected articles show that remote sensing techniques are highly efficient in mapping and monitoring soil erosion compared to traditional methods. Most satellite-based systems across multiple platforms provide a cost-free, convenient, and time-saving method for obtaining data on how eroded areas are spatial distributed (Reddy et al., 2018).

**Table 2:** Application of different remote sensing methods to monitor land degradation in the grassland biomes of sub-Saharan Africa

Reference	Study location	Objective	Remote sensing technique used	Main findings
Nzuza et al., 2021	Sekhukhune District, South Africa	Assessing and mapping land degradation using a triangulation approach	The WOCAT mapping questionnaire and remote sensing techniques	Due to overgrazing low-lying areas were moderately degraded
Dube et al., 2017	King Sabata Dalindyebo local municipality in Eastern Cape Province, South Africa	Examining the spatial and temporal variations of land degradation using Landsat series data	Multispectral Landsat	The Multispectral Landsat series dataset was highly efficient in monitoring the levels of land degradation catchments
Sepuru and Dube, 2018	Sekhukhune rural district, Limpopo Province, South Africa	Understanding the spatial distribution of eroded areas by comparing evidence from two new noncommercial multispectral sensors	Sentinel 2 and Landsat 8	Raw spectral bands and vegetation indices combination derived from the used remote sensed data and notably enhanced the accuracy of detection and mapping at (α = 0.005)
Muhoyi et al., 2023	Manyame River, in Zimbabwe	Exploring the potential of GIS and remote sensing in mapping land degradation	NDVI and GIS techniques	4.08% of the area was severely degraded, 19.80% is moderately eroded, 38.58% is severely degraded, and 37.54% of the area was not degraded
Nzuza et al., 2022	South Africa	To forecast land degradation by using Sentinel-2 data together with environmental variables	Environmental variables together with Sentinel-2	The addition of multi-seasonal high-resolution Sentinel 2 data, environmental, spectral bands, and spectral vegetation indices enhanced the overall model accuracy for all scenarios
Seutloali et al., 2016	South Africa	Assessing gully erosion along the major armoured roads	Remote sensing and GIS approach	The volume of gullies strongly influenced the hill slope gradient and roads
Taruvinga, 2009	South Africa	Use remote sensing to map gullies	TM-derived VI techniques, and SPOT 5 panchromatic imagery	The SVM classification technique was shown to be the most accurate way for mapping gullies using remote sensing Landsat TM showed the greatest potential to map gullies
Dzurume et al., 2022	Limpopo in South Africa	Using multispectral satellite data to evaluate the effects of land management methods in wetlands	multi-date Landsat images	Between 2014 and 2018, the Makuleke wetland's area decreased by 2%, while the Nylsvley wetland decreased by 3% Built-up areas increased marginally caused by the increase in the number of people and a rise in the development of infrastructure
Odhiambo et al., 2019	Makhado in Limpopo province of South Africa	Mapping landslide prone areas using remote sensing	Aerial photographs	The actions of humans, precipitation destruction of forests, gullies, land use, slope, and increased agricultural activity all contributed to the landslides in Khalavha Village
Makaya et al., 2019	South Africa	Examining the Sentinel-2 MSI sensor's ability to detect and map the spatial distribution of gullies within a community grazing environment	Sentinel-2 MSI sensor	There was no significant difference that was observed between the environmental variables across the various estimated gully volumes, with a correlation between the variables (R = 0.190526)
Watene et al., 2021	Kenya	The study examines the spatial–temporal variability of future rainfall erosivity and its effects on soil loss	RUSLE and CMIP5 models RCP	Estimated soil erosion was “4.76 t ha^−1^ yr^−1”^ The estimated increase in soil loss ranges from 29 to 60%
Wickama et al., 2018	Tanzania	To Modell and map soil erosion in smallholder agro-ecosystems	Remote sensing, GIS and soil erosion modelling models	There were significant differences in soil conditions, vegetation cover, slope, and soil losses
Igbokwe, et al., 2008	Nigeria	To map and monitor the impacts of gully erosion	USLE, Remote Sensing and GIS	The developments of gullies were more pronounced in areas with high terrain undulation
El Haj Tahir, et al., 2010	Blue Nile, Eastern Sudan	Mapping soil erosion areas	ASTER imagery, SRTM and GIS	The incision of gullies in the region was strongly linked to topography, particularly river flow networks and slope
Mhangara et al., 2012	Keiskamma catchment, South Africa	Assessing the risk of soil erosion	GIS and remote sensing	The catchment experiences soil losses of 2576 t ha^−1^ yr^−1^ or higher, while 65% experiences low to moderate levels of soil loss
Århem, & Fredén, 2014	Mara region, Tanzania	Evaluating the effect of land cover change on soil erosion	Landsat TM and ETM + images, RUSLE	Given rain fall kept average, soil erosion was influenced by land cover changes
Emeribe et al., 2002	University of Benin, located in Benin City, Nigeria	Estimating annual amount of soil loss	Landsat TM imagery, RUSLE coupled with GIS Technique	The study reveals a high annual rainfall-runoff erosivity value in the study area, with a low and uniform cover management factor throughout the Gulf site
Chuma et al., 2023	Democratic Republic of Congo	Mapping the susceptibility of Gully erosion	Machine learning, Landsat 8, and ASTER	The occurrence of gullies was significantly influenced by factors such as NDVI, slope, distance to roads, distance to rivers, and Stream Power Index
Kulimushi et al., 2021a, 2021b	Democratic Republic of Congo	Evaluating the risk of soil erosion	RUSLE, GIS and Sentinel-3 OLCI (2013–2019)	Soil erosion was estimated to be “2.084 million tons”, and an average of 138.2t ha^−1^ yr^−1^
Ewunetu et al., 2021	Blue Nile River	The study employs a spatial multicriteria evaluation technique to map and assess the status of land degradation	Landsat, ASTER Global Digital Elevation Model	Soil erosion and severe biological decline were vital indicators of land degradation in the study area
Jiang, 2013	Mount Elgon, Uganda	GIS-based time series study soil erosion risk in a micro-catchment	ASTER remotely sensed data, DMEs and RUSLE	There was less soil erosion in mountainous areas
Balabathina et al., 2019	Northwestern region of Ethiopia	Estimating soil erosion using remote sensing and universal soil loss equations	Landsat-8 Imageries, GIS, and USLE	The soil loss in a year was 8, 43,736 tons. The average soil erosion rate was higher in the lower catchment. As compared to the upper catchment. The lower record 843 736 tons and the upper records 686 705
Okenmuo & Ewemoje, 2023	Nigeria	Utilizing RUSLE, GIS, and remote sensing to estimate soil water erosion	RUSLE, remote sensing, and GIS	Soil erosion is grouped as too low “55.1% < 20 ha^−1^ y^−^, low 31.3% 21–82 ha^−1^ y^−^, medium 9.4% 82–243 t ha^−1^ y^−^, high 3% 234–543 t ha^−1^; and extreme 1.2% > 543 t ha^−1^ y^−^”,
Nambajimana et al., 2019	Rwanda	Assess water erosion in Rwanda using different techniques	MODIS and ASTER GDEM	Terracing can decrease soil loss from “14.6 t ha^−1^ y^−1^ to 11.7 t ha^−1^ y^−1”^
Harmse et al., 2022	South Africa	Examining various vegetation indices to quantify localised overgrazing	Sentinel-2 imagery	Remotely sensed data is analysed spatially to give a comprehensive overview of livestock movement patterns on a landscape-scale
Azua et al., 2020	Nigeria	Modelling of soil erosion	Landsat 8 OLI, RUSLE, and GIS	The factors that increase soil erosion in the area includes slope, low vegetation cover and high rainfall
Bouaziz et al., 2011	Ethiopia	Using aster data and geomorphologic analysis to map gullies	SRTM and ASTER DEM	This quantitative analysis generates maps that enable the monitoring and mapping of land degradation due to gully-widening
Tilahun et al., 2023	Ethiopia	To evaluate the nexus between the dynamic of land use, land cover, and soil erosion	Landsat imageries	Soil loss per year varied from 0 to 6953.23 ha^−1^ y^−1^ in 2020, with the highest levels observed in 1986, 1997, 2009, and 2020
Vrieling et al., 2006	Tanzania	Evaluating the spatial risk of soil erosion	Landsat and SRTM DEM	Data such as SRTM DEM and Landsat images provide high-quality vegetation and topographic information, enabling quantitative results on spatial variation of soil loss
Le Roux et al., 2008	South Africa	Predicting soil erosion at national scale	MODIS-derived spectral index and RUSLE	Eastern Cape and KwaZulu-Natal Provinces had highest erosional potentials in South Africa
Okou et al., 2016	Republic of Benin	Mapping soil erosion risk at a regional scale for decision support	ASTER, MODIS, GIS, LANDSAT 7 ETM + images	The study indicated that the erosion risk is very low to low in 21.8%, medium in 58.5%, and high to very high in 19.5% of the study region
Phinzi et al., 2017	Eastern Cape Province South Africa	Applying remote sensing to monitor soil erosion	Landsat8 Operational Land Imager	SAVI achieved 83% classification accuracy and 64% kappa statistics, NDVI and SARVI got 81% accuracy and 60% and 59% kappa statistics, respectively
Dondofema et al., 2008	Zhulube Meso-catchment of Zimbabwe	Evaluating the efficacy of GIS and remote sensing techniques in gully identification	Landsat TM imagery and GIS	GIS and remote sensing methods were utilized for gully identification, with accuracy varying based on the spatial, spectral, and temporal resolution of the imagery
Seutloali et al., 2017	The former South African homelands of Transkei	Assessing and mapping the severity of soil erosion	30-m Landsat multispectral satellite data	Approximately 74% rill erosion occurred in the year 1984 and 54% in 2010. Soil erosion is accelerated by steeper areas, high stream power index, lower cover of vegetation, or a low topographic wetness index of below 5%

WOCAT: World Overview Conservation Approaches and Technology, NDVI: Normalized difference vegetation index, GIS: Geographic Information Systems, SVN: The Support Vector Machine, VI: Vegetation Index, TM: Thematic Mapper, MSI: Multispectral Instrument, RUSLE: Revised Universal Soil Loss Equation, CMIP5: Coupled Model Intercomparison Project, RCP: Representative Concentration Pathway, t ha^−1^ yr^−1^: Tones per hectare per year, ASTER: Advanced Spaceborne Thermal Emission and Reflection Radiometer, SRTM: Shuttle Radar Topography Mission, DEM: Digital Elevation Model, TWI: Wetness Index, SPI: Stream Power Index

#### Spectral features (wavebands and vegetation indices)

Sepuru & Dube (2018), reported that Sentinel 2 MSI detected and mapped eroded regions with overall accuracy values slightly greater than those from Landsat 8 OLI. By integrating spectral vegetation indices with Sentinel-2 MSI spectrum reflectance information, classes of eroded surfaces were relatively well differentiated from other land cover types, and high precision in terms of ratios of OA, PA, and UA were achieved as compared to those from Landsat 8 OLI (Makaya et al., 2019). In the case of only extracted spectral data, Sentinel-2 MSI was slightly better than Landsat 8 OLI and produced similar results. The primary reason for this is due to Sentinel-2's higher spatial resolution of 10 m for visible and NIR bands compared to Landsat 8's 30 m, and its additional spectral bands, which enhance its ability to capture finer details and subtle variations in surface reflectance. Sentinel-2 MSI was outperformed by Landsat 8 OLI when spectral vegetation indices were applied or due to differences in band placement, spectral sensitivity. Overall, Sentinel-2 MSI outperformed Landsat 8 OLI, but the choice of sensor may depend on the specific application and analytical approach (Nzuza et al., 2021; Sepuru and Dube, 2018).

#### Advancement in remote sensing techniques in monitoring soil erosion: A South Africa case study

A total of 14 studies of 34 studies included in the systematic review were conducted in South Africa, indicating that the country dominates in remote sensing technique applications in monitoring soil erosion because of various factors, including the government funds to conduct environmental studies and data availability. The selected studies illustrated in Table 3 above include the study conducted using course-resolution Landsat 5 in 2009 to the use of Sentinel 2 higher resolution in 2022. This supports spectral capabilities, spatial resolution, and modelling accuracy increase as time increases. Soil erosion rates (t ha⁻^1^ y⁻^1^) were compared amongst these sensors to show how advancements in remote sensing have enhanced the detection and quantification of soil loss. However, from the observations, it was noted that SAR data in sub-Saharan Africa has not yet been employed to monitor soil erosion in grassland biomes. Nzuza et al. (2022), uses Google Earth Engine (GEE) because it has the potential to automate multi-temporal soil erosion assessment. Future researchers are recommended to GEE because it is used to handle huge data on a large scale. Also, SAR needs to be integrated to improve model scalability in the sub-Saharan region. Field observations were included in the table because they are used to validate the remote sensing outputs. The use of Landsat 5, which is an improved resolution, managed to capture moderate erosion estimated at 15–65 t ha⁻^1^ y⁻^1^. The use of higher resolution, which is Sentinel-2 MSI, and machine learning (Nzuza et al., 2022) used to estimate soil erosion rates of estimates (5–35 t ha⁻^1^ y⁻^1^). The use of Landsat managed to produce inputs used for RUSLE to predict gully and rill soil loss (Phinzi et al., 2017; Taruvinga, 2009.). The use of Sentinel-2’s spectral indices as inputs of soil erosion models and they were used to detect overgrazing.

**Table 3:** Application optical remote sensing methods to monitor soil erosion: A South Africa case study

Reference	Study location	Sensor used	Erosion rate (t ha^−1^ y^−1^)	Field observations	Remote sensing key inputs	Erosion models	GEE
Taruvinga, 2009	KwaZulu Natal	Landsat 5 (TM)	Not quantified	Large gullies were visible	NDVI	NDVI and visual interpretation	Not used
Mhangara et al., 2012	Keiskamma Catchment	Landsat 5(TM)	5–50	High erosion on steep slopes and bare soil	NDVI, Slope (DEM), land cover	RUSLE and GIS	Not used
Phinzi et al., 2017	Eastern Cape	Landsat 8	10–60	Gully erosion correlated with slope	NDVI, Rainfall (R-factor), Slope	USLE	Nott used
Seutloali et al., 2017	The former South African homelands of Transkei	30-m Landsat multispectral satellite data	15–70	Severe erosion in degraded grasslands	NDVI, SBI Slope,	RUSLE	Not used
Harmse et al., 2022	South Africa	S entinel-2 (MSI)	Not quantified (overgrazing severity classes)	Overgrazing linked to erosion vulnerability	NDVI, SAVI, EVI	Vegetation Index Thresholds	Not used
Nzuza et al., 2022	Greater Sekhukhune District	Sentinel-2 (MSI)	Predicted risk classes	Land degradation hotspots were identified	NDVI, SAVI, Spectral Indices (BSI, CI)	Machine Learning (RF), NDVI SAVI	GEE was used

TM: Thematic Mapper, OLI: Operational Land Imager, MSI: Multispectral Instrument, NDVI: Normalized Difference Vegetation Index, SAVI: Soil-Adjusted Vegetation Index, BSI: Bare Soil Index, CI: Clay Index, TPI: Topographic Position Index, DEM: Digital Elevation Model, USLE: Universal Soil Loss Equation, RUSLE: Revised Universal Soil Loss Equation, ML: Machine Learning, RF: Random Forest, GEE: Google Earth Engine, DEM: Digital elevation model, SBI: Soil Brightness Index, EVI: Enhanced Vegetation Index

#### Earth observation sensors

El Haj Tahir et al. (2010), reported out that STER-derived DEMs in Eastern Sudan's Blue Nile are less accurate than SRT due to low optical contrast in rural areas, shows the increase in the application use of remote sensing. The transition from traditional monitoring methods to aerial images and satellite-borne sensors has significantly improved the grassland biome research community. MODIS was launched in 2000 and resulted in an increase in remote field sensing, hence increasing the frequency of publications on monitoring soil erosion in grassland occurring in the 2000 s (Makaya et al., 2019; Sepuru and Dube, 2018). The launch of Sentinel 2 MSI and Landsat 8 OLI in the twentieth century increased the number of publications on monitoring soil erosion because they offered free access to data. Numerous studies explore the use of SAR sensors to estimate soil erosion progression in grassland biomes, favoured by researchers for its ability to select suitable wavelengths (Rawat et al., 2016).

#### Gully and landslides development in grassland biomes

Studies on gullies were primarily conducted in Limpopo, the Eastern Cape, and KwaZulu-Natal Province (Dube et al., 2017; Le Roux et al., 2008; Nzuza et al., 2021; Odhiambo et al., 2019; Sepuru and Dube, 2018). Numerous studies have been conducted in these provinces, and this highlights the significance of gullies as a key focus of research in the areas. Overgrazing contributes to soil loss in grassland biomes as shown in Table 2 (Nzuza et al., 2021). However, gully developments are more pronounced in areas with high terrain undulation. Gully incisions are linked to topography, particularly river flow networks and slopes, and climate change. These can trigger new processes due to increased rainfall or weakened land cover. The selected articles also report that most grasslands were moderately eroded. The reports included in the study recommend monitoring erosion and implementing sustainable practices to reduce soil loss.

Furthermore, the findings indicated that the development of gullies in grasslands varies because of different factors, which include vegetation density, slope, climate, and TWI. In support of that, one of the included studies reports “no significant difference between the environmental variables across the different estimated gully volumes and indicates a weak correlation between the variables of R = 0.19” (Makaya et al., 2019). The findings show that gully developments occur on gentle slopes of approximately 7^0^ (Makaya et al., 2019). Field observations were used in selected studies to validate the conclusions from remote sensing. Gully-prone areas are often linked to convergence zones with concave platform curvature, triggering gullies due to increased runoff volume downslope (Phinzi & Ngetar, 2017).

The reports indicated that population growth accelerates the depletion of environmental resources, leading to higher deforestation rates, which in turn increases landslide activity in mountainous areas and causes soil erosion in grassland biomes (Odhiambo et al., 2019). In grasslands, landslides are due to slope saturation by water. The water effect is a result of intense rainfall, groundwater levels, and changes along coastlines, earth dams, and riverbanks (Popescu, 2002).

#### Variation in the rate of soil erosion in different agro-ecosystems

In Kenya, the estimated soil loss was 4.76 t ha^−1^ yr^−1^, expected to rise by 29–60% (Watene et al., 2021). The factors causing an increase in soil loss include climate change, drought, illegal logging, aggressive agricultural practices, and cattle overgrazing. Report by Watene et al. (2021), indicated that natural forests have less soil loss of “1.57 t ha^−1^ yr^−1”^, and croplands are highly degraded agro-ecosystems because of uncovered soils from poor conservation and cultivation methods and practices. Croplands’ topsoil is exposed in off-seasons and often blown away by wind or rain. Dense forest plants keep soil bound in locks of roots, thus preventing erosion. In Kenya, agro-ecological ecosystems showed “significant differences (p < 0.05) in terms of soil” conditions, vegetation cover, slope, and soil losses (Watene et al., 2021).

### Limitations of the study

- In sub-Saharan Africa, some countries use French, Portuguese, or other languages to write their research findings. This poses a challenge when conducting the literature search since the current study only included English articles.
- Some articles were not accessible, and some were unavailable at total length due to limited institutional subscription, pay walls and restricted access. For the above reasons, it was difficult to quantify all the studies on integrating optical and SAR systems in monitoring soil erosion in sub-Saharan grassland biomes.
- Selection bias in studies can lead to incorrect evidence. PRISMA reduces this by using multiple databases, languages, and grey literature sources, and publishing search methods in a priori protocol for peer review.
- Lack of transparency or reliability is one of the limitations that were encountered. However, the authors employed PRISMA to be explicit and to maintain the quality and standard of the systematic review.

### Research gaps and future study recommendations

- Monitoring the progression of soil erosion in sub-Saharan African grassland biomes is necessary. Considerable gaps exist worldwide, specifically on the African continent, in integrating remote sensing techniques to monitor and map soil erosion progression.
- Application of multiplatform satellite-based systems in monitoring soil erosion in sub-Saharan Africa has yet to attract significant attention in many countries within the sub-Saharan Africa.
- Future researchers in sub-Saharan Africa should use remote sensing-based vegetation indices like modified NDVI, NDSI, and TCT to estimate soil erosion, as they are commonly used in developed countries.

## Conclusion

This study systematically reviewed the literature on integrating optical and SAR systems, as well as multiplatform satellite-based systems, for monitoring soil erosion in sub-Saharan grassland biomes. Through evaluation of advancements and identification of research gaps, the study highlights the growing role of remote sensing technologies in addressing soil erosion challenges. The review of 34 articles revealed that most studies were conducted in South Africa on the application of multiplatform satellite-based systems for soil erosion monitoring. The application of multiplatform satellite-based systems for monitoring and mapping soil erosion progression has yet to attract some countries within sub-Saharan Africa. Remote sensing techniques mainly employed in monitoring and mapping soil erosion include multispectral Landsat series, Sentinel 2 satellite, Landsat 8, ASTER, and SRTM. These techniques are highly efficient in mapping and monitoring soil erosion compared to traditional methods. For large-scale monitoring of soil erosion, remote sensing is highly effective because it provides extensive spatial coverage, and it can track changes over time. For small-scale studies, field measurements are essential as they offer highly accurate, localized data that can capture site-specific details. By combining these approaches, more accurate and reliable results can be produced. The use of multispectral and SAR data, particularly in combination, has the potential to provide more comprehensive and timely assessments of soil erosion, especially in cloud-prone areas where optical data alone is insufficient. However, the integration of optical and SAR data remains underexplored, representing a critical gap in current research. The study found that gully developments are more pronounced in high terrain undulation areas, strongly associated with topography, and suggests using remote sensing-based vegetation indices for soil erosion estimation.

## Data Availability

No datasets were generated or analysed during the current study.

## Declarations

All authors have read, understood, and have complied as applicable with the statement on “Ethical responsibilities of Authors” as found in the Instructions for Authors. All ethical practices have been followed in relation to the development, writing, and submission of the manuscript.
